# HbA_1c_ is associated with altered expression in blood of cell cycle- and immune response-related genes

**DOI:** 10.1007/s00125-017-4467-0

**Published:** 2017-11-20

**Authors:** Roderick C. Slieker, Amber A. W. A. van der Heijden, Nienke van Leeuwen, Hailiang Mei, Giel Nijpels, Joline W. J. Beulens, Leen M. ’t Hart

**Affiliations:** 1 Department of Molecular Cell Biology, Leiden University Medical Center, Postal Box 9600, 2300 RC Leiden, the Netherlands; 2 Department of Epidemiology and Biostatistics, Amsterdam Public Health Research Institute, VU University Medical Center, Amsterdam, the Netherlands; 3 Department of General Practice and Elderly Care Medicine, Amsterdam Public Health Research Institute, VU University Medical Center, Amsterdam, the Netherlands; 4 Sequencing Analysis Support Core, Leiden University Medical Center, Leiden, the Netherlands; 5 Julius Center for Health Sciences and Primary Care, University Medical Center Utrecht, Utrecht, the Netherlands; 6 Molecular Epidemiology Section, Leiden University Medical Center, Leiden, the Netherlands

**Keywords:** Blood, Gene expression, Glucose levels, HbA_1c_, Immune response, RNA sequencing

## Abstract

**Aims/hypothesis:**

Individuals with type 2 diabetes are heterogeneous in their glycaemic control as tracked by blood HbA_1c_ levels. Here, we investigated the extent to which gene expression levels in blood reflect current and future HbA_1c_ levels.

**Methods:**

HbA_1c_ levels at baseline and 1 and 2 year follow-up were compared with gene expression levels in 391 individuals with type 2 diabetes from the Hoorn Diabetes Care System Cohort (15,564 genes, RNA sequencing). The functions of associated baseline genes were investigated further using pathway enrichment analysis. Using publicly available data, we investigated whether the genes identified are also associated with HbA_1c_ in the target tissues, muscle and pancreas.

**Results:**

At baseline, 220 genes (1.4%) were associated with baseline HbA_1c_. Identified genes were enriched for cell cycle and complement system activation pathways. The association of 15 genes extended to the target tissues, muscle (*n* = 113) and pancreatic islets (*n* = 115). At follow-up, expression of 25 genes (0.16%) associated with 1 year HbA_1c_ and nine genes (0.06%) with 2 year HbA_1c_. Five genes overlapped across all time points, and 18 additional genes between baseline and 1 year follow-up. After adjustment for baseline HbA_1c_, the number of significant genes at 1 and 2 years markedly decreased, suggesting that gene expression levels in whole blood reflect the current glycaemic state and but not necessarily the future glycaemic state.

**Conclusions/interpretation:**

HbA_1c_ levels in individuals with type 2 diabetes are associated with expression levels of genes that link to the cell cycle and complement system activation.

**Electronic supplementary material:**

The online version of this article (10.1007/s00125-017-4467-0) contains peer-reviewed but unedited supplementary material, which is available to authorised users.

## Introduction

Individuals with type 2 diabetes are heterogeneous in their disease trajectory, glycaemic control over time [1], response to therapy and in the disease-related complications they develop, including micro- and macrovascular complications [2]. Poor glycaemic control has been associated with a higher incidence of developing microvascular complications [1, 3]. Therefore, individuals with type 2 diabetes would benefit from new markers for future glycaemic control, especially when in an early stage of the disease.

Much effort has been spent identifying common gene variants that mark disease risk and progression, but genetic variants contribute little in addition to classic risk factors, especially in people below 50 years of age [4]. In addition, genetic risk scores explain only 10–15% of the heritability of type 2 diabetes [5]. Accelerated by recent technological advances, other molecular variables, such as epigenetic modifications and gene expression, are increasingly being investigated in relation to blood glucose and type 2 diabetes and its progression. For example, DNA methylation near known type 2 diabetes loci (for example, *KLF14*, *ZNF518B*, *INS*) is associated with measures of glucose homeostasis (HbA_1c_, 2 h insulin) in healthy individuals [6, 7].

At the transcriptional level, early studies have found multiple genes to be differentially expressed between the control group and individuals with (pre)diabetes in target tissues [8–11], and also in blood [12–15]. Using a genome-wide approach in peripheral blood mononuclear cells (PBMCs), genes from the c-Jun N-terminal kinase (JNK) and oxidative phosphorylation pathways were differentially expressed in individuals with and without type 2 diabetes [12]. In addition to case–control designs, a limited number of studies have also investigated links between gene expression in blood and target tissues and glycaemic control and disease-related complications. In PBMCs, the expression of genes encoding TNF-α and IL-6 was elevated in individuals with type 2 diabetes with micro- (*n* = 29) and macroalbuminuria (*n* = 31) compared with the control group (*n* = 22) and individuals with type 2 diabetes and normoalbuminuria (*n* = 18) [13]. In the same study, *TNF* expression correlated with HbA_1c_ levels [13].

While there are indications that measures of glycaemic control are reflected in molecular measures and blood is an interesting tissue from an etiological perspective, the number of studies that have investigated the relationship between gene expression in blood and disease progression is limited. Those that have been conducted have tended to be small cross-sectional case–control studies. We have investigated the relationship between blood gene expression levels and HbA_1c_ levels in almost 400 individuals with type 2 diabetes selected from the Hoorn Diabetes Care System (DCS) cohort [16].

## Methods

### Study population

Individuals who participated in this study are part of the Hoorn DCS cohort, a prospective cohort of over 12,000 individuals with type 2 diabetes [16]. People visit the DCS annually for routine care and data collection, including anthropometric, fasting glucose, HbA_1c_, blood lipid and blood pressure measurement and information on medication use. A subset of the individuals in the Hoorn DCS cohort are part of a biobank in which biological material is stored for research purposes. Blood RNA was collected in 2013 and 2014 from 1033 individuals who had participated in the biobank previously, without any specific selection criteria; this group were representative of the individuals who visited DCS in 2013 (ESM Table 1). From this group of 1033, we selected 400 individuals (ESM Table 1) based on the following criteria: age at onset between 40 and 75 years; European descent; diabetes duration less than 10 years; and estimated (e)GFR > 30 ml/min. Untreated individuals were excluded. Each participant gave informed consent and the study was conducted in line with the Declaration of Helsinki.

### RNA sequencing

Blood for RNA was collected in Tempus tubes (ThermoFisher Scientific, Waltham, MA USA), and RNA was isolated from whole blood using the Direct-zol RNA MiniPrep (Zymo Research, Irvine, CA USA). RNA concentrations were determined using Nanodrop (Nanodrop, Wilmington, DE USA) and, in a subset, RNA integrity was examined using lab-on-a-chip (Agilent, Santa Clara, CA, USA). Whole-genome transcriptome data were generated at the human genotyping facility (HugeF) of the Erasmus Medical Center (the Netherlands, www.glimdna.org). RNA sequencing libraries were generated using the Illumina Truseq v2 library preparation kit (Illumina, San Diego, CA, USA). Libraries were paired-end sequenced (50 bp) using the Illumina Hiseq2000.

Samples (*n* = 44) with a library size smaller than 30 million reads were re-sequenced and the libraries of the first and second run were combined*.* Reads passing the chastity filter were combined in sets with Illumina’s CASAVA. Raw read quality was assessed using FastQC (v0.10.1) [17]. The adaptors identified by FastQC were clipped using Cutadapt (v1.1) using default settings [18]. To trim low-quality ends of the reads, Sickle (v1.2) was used (minimum length 25, minimum quality 20) [19]. Reads were aligned to the genome using STAR (v2.3.0) [20].

To avoid reference mapping bias, SNPs in the Dutch population (Genome of the Netherlands [GoNL]) with minor allele frequency (MAF) > 0.01 in the reference genome were excluded. Read pairs with eight mismatches at most, mapping to five positions at most, were used. Mapping statistics from the binary alignment map files were acquired through Samtools flagstat (v0.1.19-44428cd). The 5′ and 3′ coverage bias, duplication rate and insert sizes were assessed using Picard tools (v1.86). Gene expression, as read count per gene, was calculated using htseq (v0.6.1p1) with default settings based on Ensembl v71 annotation (corresponding to GENCODE v16) [21]. Gene counts were normalised for GC content and gene length using the R package cqn [22]. To exclude sample mix-ups, genotypes of 50 frequently occurring SNPs were called and compared with available genotype data. Sex was confirmed using gene expression of *XIST* (chromosome X) and *UTY* (chromosome Y). Genes with ≤5 reads in ≥75% of the samples were discarded, as were genes on the sex chromosomes. The final dataset comprised gene expression levels of 391 individuals comprising 15,564 autosomal genes.

### Models with blood HbA_1c_

HbA_1c_ was measured using a turbidimetric inhibition immunoassay (Cobas c501, Roche Diagnostics, Mannheim, Germany). All analyses between gene expression and HbA_1c_ at baseline, and 1 or 2 year follow-up were performed using generalised linear models, implemented in the R package edgeR [23]. HbA_1c_ levels were log transformed as they were not normally distributed. The model was adjusted for sex, age, BMI, blood cell composition, metformin dose, sulfonylurea and/or insulin use and technical covariates, as these are factors known to influence gene expression levels and/or HbA_1c_ levels.

In an extended model, additional factors were added including systolic blood pressure, education level (low, mid, high) and smoking status (non-smoker, former, current). Blood cell counts were determined with a UniCel DxH 800 Coulter Cellular Analysis System (Beckman Coulter) and the FC 500 Series system (Beckman Coulter, Brea, CA, USA). Blood cell fractions were also estimated using the R package wbccPredictor [24]. The imputed cell fractions showed a strong correlation with the measured counts (ESM Fig. 1). Blood cell fractions are strongly correlated with each other; therefore, five principal components were included in the model to adjust for the effect of blood cell composition. To investigate the effect of baseline HbA_1c_ on the association at follow-up, we also added baseline HbA_1c_ to the model for the 1 and 2 follow-up in addition to all the other covariates described above. The effect of medication was assessed by performing the model on metformin users only (*n* = 252), excluding individuals with other forms of (mono/dual) therapy. In the case of missing data or loss at follow-up, the models were performed only with individuals with complete data. The *p* values for all generalised linear models of the 15,564 genes (15,564 tests) were false-discovery rate (FDR) adjusted using the Benjamini–Hochberg procedure as implemented in the *p.adjust* function in R. A FDR-adjusted *p* value below 0.05 was considered significant.

### Co-expression networks

Co-expressed genes (with expression profiles showing a high correlation, suggesting a functional relationship between the genes) were identified using mixed-model co-expression on log-transformed reads per kilobase million (RPKM) values [25]. Mixed-model co-expression is an R-implemented method that uses Pearson correlation while adjusting for confounding, thereby excluding spurious correlations. The method is described in more detail in Furlotte et al. [25]. Genes were considered co-expressed when the absolute correlation was higher than 0.3 with a *p* value ≤ 0.001. Clusters within the gene co-expression network (i.e. those with a high number of correlated genes) were identified using Cytoscape v3.4.0. Co-expression of genes was plotted using the R package edgebundleR [26]. Graphs were produced using the R package ggplot2 [27].

### Gene set enrichment

Genes within the three co-expressed clusters were tested for over-representation in gene sets using the default settings of REACTOME (V61) [28]. Pathways with *p*_FDR_ < 0.05 were considered significant.

### eQTLs

A public expression Quantitative Trait Locus (eQTL) database was used (www.genenetwork.nl/biosqtlbrowser/, accessed July 2017) to identify SNPs that influence gene expression [29]. Genes were mapped to associating SNP based on the Ensembl gene ID. Diabetes-related traits were obtained from the genome-wide association study (GWAS) catalogue and the MAGIC GWAS [30]. The Venn diagram was created using jVenn.

### External data

Genes identified at baseline were investigated in the target tissues muscle and pancreas, from two external datasets. The first external dataset consisted of gene expression levels in pancreatic islets measured with the Affymetrix Human Gene 1.0 ST Array (Gene Expression Omnibus [GEO] accession number GSE54279), comprising 113 individuals with HbA_1c_ in the range 23.5–85.8 mmol/mol (4.3–10%) and median 39.9 mmol/mol (5.8%) [31].

The second external dataset consisted of gene expression levels in muscle, accessed with the Affymetrix GeneChip Human Genome U133 Plus 2.0 Array (GEO accession number GSE18732), comprising 115 individuals with HbA_1c_ range 33.3–136.1 mmol/mol (5.2–14.6%) and median 39.9 mmol/mol (5.8%) with and without type 2 diabetes [32].

Both datasets included expression at the transcript level rather than the gene level. To make the datasets comparable, the average expression of all transcripts of a gene was calculated for 99 genes that could be retrieved in both datasets (out of the 220 genes, 45%) that were present in both datasets. HbA_1c_ levels were converted to International Federation of Clinical Chemistry and Laboratory Medicine (IFCC) HbA_1c_ levels and log transformed. The associations between HbA_1c_ levels and gene expression in muscle and pancreatic islets were determined using Pearson correlation.

## Results

Individual characteristics at baseline and follow-up are given in Table 1. Individuals selected for RNA sequencing were a representative subset of all individuals with blood RNA, the entire cohort and the biobank subset, as their characteristics were very similar (ESM Table 1). Diabetes duration was one of the selection criteria and this was shorter in the group of individuals with RNA sequencing compared with the entire cohort and the biobank subset (ESM Table 1).

**Table 1:** Individual characteristics of the sample of the DCS cohort

Characteristic	Baseline (*n* = 391)	1 year follow-up (*n* = 372)	2 year follow-up (*n* = 362)
Sex (% female)	41.2	41.7	41.2
Metformin use (%)	89.5	87.9	89.2
SU use (%)	11.8	15.9	20.4
Insulin use (%)	12.0	12.6	14.9
Age (years)	64.0 (57.3, 70.0)	64.9 (58.3, 71.0)	66.4 (59.4, 72.0)
Diabetes duration (years)	3.7 (2.1, 5.5)	4.6 (3.1, 6.5)	5.6 (4.2, 7.7)
Glucose (mmol/l)	7.9 (7.2, 9.1)	8.0 (7.0, 9.2)	8.1 (7.2, 9.5)
HbA_1c_ (%)	6.4 (6.0, 7.0)	6.5 (6.1, 7.2)	6.6 (6.2, 7.3)
HbA_1c_ (mmol/mol)	47 (42, 53)	48 (44, 55)	49 (44, 56)
BMI (kg/m^2^)	29.5 (26.4, 33.0)	29.3 (26.4, 32.7)	29.2 (26.4, 33.0)
LDL-cholesterol (mmol/l)	2.3 (1.8, 2.9)	2.2 (1.8, 2.8)	2.2 (1.7, 2.9)
HDL-cholesterol (mmol/l)	1.2 (1.0, 1.5)	1.2 (1.0, 1.5)	1.2 (1.0, 1.4)
Triacylglycerol (mmol/l)	1.6 (1.1, 2.2)	1.5 (1.1, 2.2)	1.6 (1.1, 2.2)
Systolic BP (mmHg)	134 (124, 152)	138 (126, 151)	136 (126, 151)
Diastolic BP (mmHg)	80 (75, 85)	80 (74, 85)	79 (74, 85)
eGFR (ml/min)	85.8 (73.4, 98.5)	84.4 (71.3, 95.9)	83.7 (70.8, 95.2)

Data are presented as median (first quartile, third quartile) unless otherwise indicated

SU, sulfonylurea

**Fig. 1:**
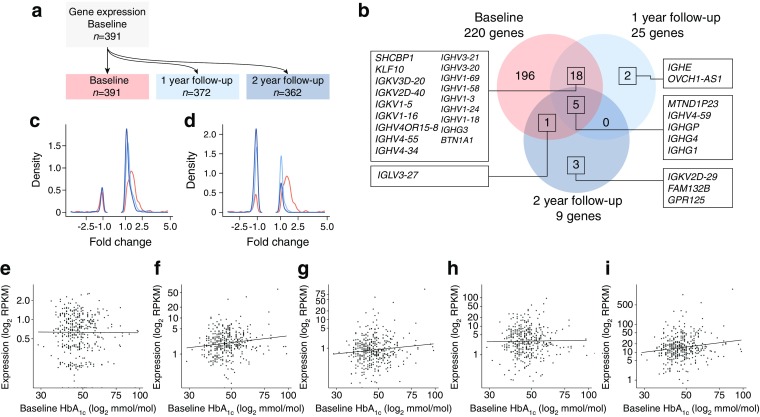
Association between gene expression levels and HbA_1c_ levels in whole blood. (**a**) Experimental setup. (**b**) Overlap between genes identified as associated with HbA_1c_ at baseline, and at 1 and 2 year follow-up. (**c**, **d**) Density of fold change at baseline (pink), 1 year (light blue) and 2 year (dark blue) follow-up of genes associated with baseline HbA_1c_ levels without adjustment for baseline HbA_1c_ (**c**) or with adjustment for baseline HbA_1c_ (**d**). (**e–i**) Scatterplot of gene expression levels against baseline HbA_1c_ for the five genes identified at each of the follow-up time points: *MTND1P23* (**e**), *IGHV4*–*59* (**f**), *IGHGP* (**g**), *IGHG4* (**h**) and *IGHG1* (**i**). Data presented are unadjusted for covariates in the model. To convert values for HbA_1c_ in mmol/mol into %, multiply by 0.0915 and add 2.15. GPR125 is also known as *ADGRA3*

Gene expression levels were tested for an association with HbA_1c_, a measure of glucose levels over the preceding weeks, at baseline and 1 and 2 year follow-up (Fig. 1a). Of the 15,564 genes that passed quality control, 220 genes (1.4%) were associated (*p*_FDR_ ≤ 0.05) with HbA_1c_ levels at baseline with adjustment for covariates. Of these, the majority (183 genes) were upregulated (fold change, 1.05–4.80; ESM Table 2) and 37 genes were downregulated (fold change, 1.05–3.34). Blood cell fractions were both measured and estimated based on the gene expression data, but there was no difference in the magnitude of the effect with measurements vs estimates (ESM Fig. 2a). In addition, the observed associations were not driven by differences in medication usage as: (1) all genes showed the same direction of effect in a stratified analysis with metformin users only (*n* = 252, ESM Fig. 2b); and (2) the effect sizes (log fold change) of the models with and without adjustment for medicine usage were highly correlated (ESM Fig. 2c). To investigate the effect of other factors, such as lifestyle, we extended our model to include systolic blood pressure, education and smoking, but found no difference in the direction or magnitude of effect (*r* = 0.98, *p* < 0.00001).

The number of genes associated with HbA_1c_ at baseline was considerably higher than at 1 and 2 year follow-up, with 25 genes at 1 year (23 genes upregulated, fold change = 1.17–3.10; two genes downregulated, fold change = 2.33–2.90; ESM Table 2) and nine genes at 2 year follow-up (six genes upregulated, fold change = 1.86–2.69; three genes downregulated, fold change = 1.39–3.88). To identify genes that showed a consistent association with HbA_1c_ over time, the overlap between the three gene sets was determined (baseline and 1 and 2 years; Fig. 1b). Five genes (2.3%) were found to overlap at baseline and both follow-up time points (Fig. 1b,d); 18 additional genes were identified as overlapping at baseline and 1 year follow-up, with no genes overlapping between 1 and 2 year follow-up (Fig. 1b).

As HbA_1c_ levels across years are correlated, particularly between successive years (baseline against 1 year, *r* = 0.76, and 1 year vs 2 year, *r* = 0.74; ESM Fig. 3), we next ran the model while adjusting for baseline HbA_1c_. Of the 25 genes identified, only one remained significantly associated after 1 year follow-up (*NDN,* fold change = −2.11, *p*_FDR_ = 0.02) and two genes after 2 year follow-up (*FAM132B* [also known as *ERFE*], fold change = −1.90, *p*_FDR_ = 0.03, and *MTND1P23*, fold change = −3.55, *p*_FDR_ = 0.04). Moreover, the fold change in the association of genes identified at baseline was largely the same over time without adjustment for baseline HbA_1c_, but strongly decreased when baseline HbA_1c_ was included in the model (Fig. 1c).

### HbA_1c_-associated genes are involved in the cell cycle and immune response

Next, we explored the genes associated with HbA_1c_ at baseline in more detail. First, we investigated whether HbA_1c_ levels causally influence gene expression using Mendelian randomisation. For this, we selected 188 SNPs associated with HbA_1c_ in healthy control individuals from the MAGIC GWAS to serve as genetic instruments [30]. However, when we tested the validity of these genetic instruments in our own data, they did not pass the quality threshold (*F* value > 10), excluding the possibility of Mendelian randomisation.

Next, we investigated whether identified genes were causally related to the development of type 2 diabetes. Using a public blood eQTL database [29], we identified the 230 strongest associating SNPs near 124 genes (out of the 220). We compared these SNPs to known diabetes-related traits, but found no overlap (ESM Fig. 4). This suggests that there is no relation between known variants involved in type 2 diabetes development and the genes found in this study.

**Fig. 2:**
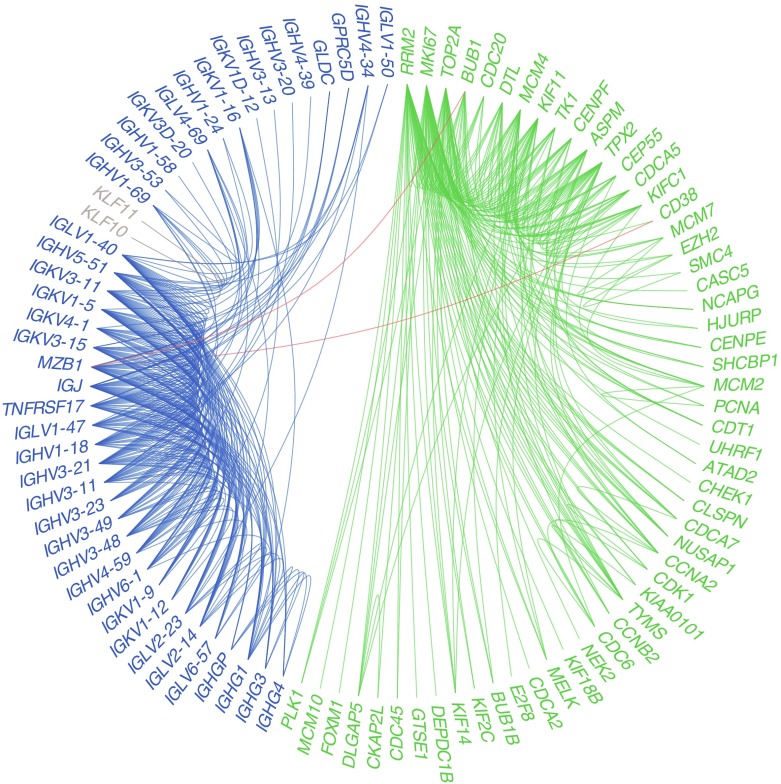
Co-expression between genes associated with HbA_1c_. Blue, gene cluster containing immune-related genes; green, gene cluster containing cell cycle-related genes; red, gene co-expression between gene clusters; grey, gene cluster containing two genes from the KLF gene family. *CASC5* is also known as *KNL1*; *KIAA0101* is also known as *PCLAF*; and *IGJ* is also known as *JCHAIN*

However, several of the 220 genes were found to have a known link to diabetes, including *CD38*, *INSR* and *PC*. *CD38* (fold change = 1.44) is a surface marker associated with insulin resistance in diabetes via the release of inflammatory cytokines [33]. *INSR* (fold change = −1.13, *p*_FDR_ = 0.03) encodes the insulin receptor important for insulin action. *PC* (fold change = 1.30, *p*_FDR_ = 5.1 × 10^−3^) encodes pyruvate carboxylase, which is involved in gluconeogenesis. To more systematically explore the relation between the genes identified, we determined whether they are co-expressed, i.e. whether they are correlated, suggesting a functional link (mixed-model co-expression, |*r*| ≥ 0.3, *p* ≤ 0.001). Co-expression was found for 99 genes (Fig. 2a), among which three clusters could be distinguished: the largest comprising 55 genes; a second smaller cluster comprising 42 genes; and the third consisting of two genes. The largest cluster showed strong over-representation in cell cycle (checkpoint) pathways (33 genes [15.0%], *p*_FDR_ = 1.33 × 10^−14^; Fig. 2 and Table 2). The second cluster showed over-representation for complement system activation and B cell signalling pathway (29 genes [13.2%], *p*_FDR_ = 1.11 × 10^−16^; Fig. 2 and Table 2), in line with the large number of genes identified that encode immunoglobulin constituents. The third cluster comprised *KLF10* and *KLF11*, which both link to cell cycle regulation.

**Table 2:** Enrichment of co-expressed gene clusters in REACTOME pathways

Cluster	Pathway identifier	Pathway name	No. genes	No. total	*P*_FDR_
1	R-HSA-173623	Classic antibody-mediated complement activation	29	98	1.11 × 10^−16^
	R-HSA-2029481	FCGR activation	29	104	1.11 × 10^−16^
	R-HSA-5690714	CD22-mediated BCR regulation	22	73	1.11 × 10^−16^
	R-HSA-2029485	Role of phospholipids in phagocytosis	29	130	1.11 × 10^−16^
	R-HSA-983695	Antigen activates BCR leading to generation of second messengers	22	110	1.11 × 10^−16^
2	R-HSA-69278	Cell cycle, mitotic	33	533	1.33 × 10^−14^
	R-HSA-1640170	Cell cycle	36	645	1.33 × 10^−14^
	R-HSA-453279	Mitotic G1–G1/S phases	14	147	7.13 × 10^−13^
	R-HSA-69620	Cell cycle checkpoints	15	188	7.13 × 10^−13^
	R-HSA-69206	G1/S transition	13	123	1.22 × 10^−12^
	R-HSA-68877	Mitotic prometaphase	13	136	3.57 × 10^−12^

FCGR, Fc-gamma receptors; BCR, B cell receptor; no. number; Cluster 1 corresponds to blue genes in Fig. 2; Cluster 2 corresponds to green genes in Fig. 2

### Expression of a subset of genes in muscle and pancreas also associates with HbA_1c_

**Fig. 3:**
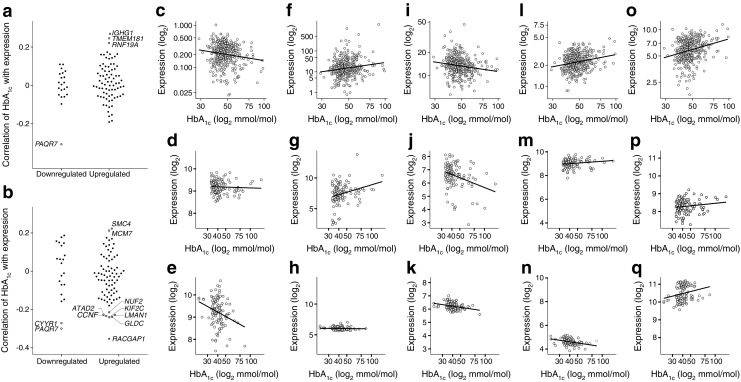
Association between HbA_1c_ levels and gene expression in muscle and pancreas. (**a**, **b**) Correlation between HbA_1c_ and gene expression for in-blood up- and downregulated genes in muscle. (**c**–**e**) HbA_1c_ against gene expression of *CYYR1*: blood (**c**), fold change = −1.41, *p* = 1.7 × 10^−2^; muscle (**d**), *r* = −0.05, *p* = 0.60; pancreas (**e**), *r* = −0.27, *p* = 3.7 × 10^−3^. (**f**–**h**) HbA_1c_ against gene expression of *IGHG1*: blood (**f**), fold change = 4.80, *p* = 7.25 × 10^−9^; muscle (**g**), *r* = 0.27, *p* = 3.6 × 10^−3^; pancreas (**h**) *r* = −0.05, *p* = 0.62. (**i**–**k**) HbA_1c_ against gene expression of *PAQR7*: blood (**i**), fold change = −1.24, *p* = 0.01; muscle (**j**), *r* = −0.31, *p* = 8.2 × 10^−4^; pancreas (**k**), *r* = −0.30, *p* = 1.3 × 10^−3^. (**l**–**n**) HbA_1c_ against gene expression of *RACGAP1*: blood (**l**), fold change = 1.11 *p* = 0.04; muscle (**m**), *r* = 0.17, *p* = 6.4 × 10^−2^; pancreas (**n**), *r* = −0.35, *p* = 1.3 × 10^−4^. (**o–q**) HbA_1c_ against gene expression of *SMC4*: blood (**o**), fold change = 1.13, *p* = 0.05; muscle (**p**), *r* = 0.14, *p* = 0.13; pancreas (**q**), *r* = 0.22, *p* = 1.8 × 10^−2^. *r*, Pearson’s correlation coefficient. To convert values for HbA_1c_ in mmol/mol into %, multiply by 0.0915 and add 2.15

To investigate whether the association with HbA_1c_ extends to target tissues, we expanded the analysis to two external datasets of muscle (*n* = 115, GSE18732) and pancreatic islets (*n* = 113, GSE54279) [31, 32]. Of the 220 genes identified at baseline, 99 (45%) were identified in both microarray-based datasets. Of the 37 genes downregulated in blood, several showed a correlation with HbA_1c_ in the same direction (*r* < −0.2, *p* ≤ 0.05; Fig. 3a,b) and PAQR7 in both target tissues (*r*_muscle_ = −0.31, *r*_pancreas_ = −0.30, *p* < 0.002; Fig. 3a,b,i–k). Among the 220 upregulated genes in blood, five genes were also found to be upregulated in target tissues: *IGHG1*, *TMEM181* and *RNF19A* in the muscle and *SMC4* and *MCM7* in the pancreas (Fig. 3a,b). Seven genes showed a correlation in the opposite direction (*r* < −0.2, *p* ≤ 0.02): *ATAD2*, *CCNF*, *NUF2*, *KIF2C*, *LMAN1*, *GLDC* and *RACGAP1* (Fig. 3a,b). Plots for the five genes showing the strongest correlations in muscle or pancreas are shown in Fig. 3c–q. For muscle, we combined data for individuals with normal glucose tolerance, impaired glucose tolerance and type 2 diabetes. However, when the analysis was performed on individuals with type 2 diabetes only (*n* = 44, 39%), similar correlations were observed compared with the analysis in all individuals (*r* = 0.54, *p* = 4.9 × 10^−9^).

## Discussion

In the current study, we investigated the relationship between gene expression levels in whole blood and HbA_1c_ in 391 individuals. The highest number of genes were associated with baseline HbA_1c_; much lower numbers were associated with HbA_1c_ level at follow-up. The direction of the effect was very similar across the different time points, although a decrease in effect size was observed with time. After adjustment for baseline HbA_1c_, most correlations of genes with follow-up HbA_1c_ lost significance. Genes identified at baseline were enriched for cell cycle and immune pathways.

Baseline HbA_1c_ was associated with 220 genes, but the number of genes strongly decreased over time, with only nine genes associated with HbA_1c_ at 2 years follow-up. This suggests that some genes reflect the HbA_1c_ levels at baseline, but not necessarily future HbA_1c_. The diminishing relationship was also seen when the genes associated at baseline with HbA_1c_ were followed across time, as fold change of association decreased with time. Moreover, the association with follow-up HbA_1c_ was driven largely by the correlation between HbA_1c_ levels across time; when adjusted for baseline HbA_1c_, the number of genes associated with follow-up HbA_1c_ further declined.

Our results give insight into the groups of genes that show aberrant expression with different HbA_1c_ levels. We identified three gene clusters as being differentially expressed: one that linked to cell cycle processes, one to immune response and the third consisted of only *KLF10* and *KLF11*. *KLF11* has been described in type 2 diabetes physiology, but has shown mixed results in GWAS [34–36]. A role for the immune system in type 2 diabetes and obesity is increasingly recognised [37, 38], making blood—in addition to target tissues like pancreas and muscle—a relevant tissue to investigate in diabetes. In healthy individuals, exposure to an OGTT leads to changes in expression of immune-related genes over a 2 h period [39]. Moreover, several blood cell types have been suggested to play a role in, for example, insulin resistance [37, 40, 41]. However, the link between the immune system and type 2 diabetes remains complex and controversial. For example, in a Mendelian randomisation study no causal links were found between IL-1 receptor antagonist (IL-1Ra) or C-reactive protein (CRP) and diabetes-related outcomes [42, 43], while IL-1Ra is associated with 2 h glucose and insulin sensitivity [44].

In case–control studies, it has been shown that type 2 diabetes is associated with altered expression of inflammatory and cell cycle genes [12, 14]. Our study suggests that, in addition to having diabetes, the level of glycaemic control is associated with immune- and cell cycle-related alterations in gene expression. Changes in gene expression in other tissues, such as lymph vessels, have also been identified and point to a role of the immune system in diabetes [45]. We also identified genes that were not only associated in blood with HbA_1c_ but also in the muscle and pancreas. Of the genes inversely associated with HbA_1c_ levels, *PAQR7* was downregulated in all three tissues. *PAQR7* is a progesterone receptor that, when activated, promotes glucose tolerance in the mouse GLUTag cell line [46].

In addition to the immune-related genes, we identified genes related to cell cycle and its checkpoints. Six of the cell cycle genes were also confirmed to have a relationship with HbA_1c_ in the pancreas in the same (i.e. *SMC4* and *MCM7*) or opposite direction (*ATAD2*, *CCNF*, *NUF2* and *KIF2C*). Dysregulation of the cell cycle in pancreas and kidneys has been described and linked to a higher risk of developing type 2 diabetes and complications in rodents [47–49]. In humans, SNPs near the cell cycle genes *CDC123* and *CDKN2A* have been found to be associated with increased susceptibility to type 2 diabetes. This suggests that high blood glucose is associated with dysregulation of the cell cycle not only in the pancreas, but also in other tissues.

A limitation of our study is the relatively heterogeneous population of individuals with type 2 diabetes. Individuals have different diabetes histories and use a variety of drugs, including drugs to control their glucose levels. Yet the heterogeneity of individuals is also part of the question, and a biomarker should be independent of a confounding effect of treatment. As the majority of individuals were taking metformin and this drug is dose-dependently associated with HbA_1c_ [50], we adjusted for the metformin dosage and for use of sulfonylureas and insulin (in addition to classic confounders such as sex, age and BMI). However, while we did not observe an effect for differences in, for example, glucose-lowering medication, education, smoking, blood pressure or BMI, it remains a limitation of our study that there may be other factors related to, for instance, lifestyle and concurrent diseases that may have affected HbA_1c_ and gene expression. A second limitation is that we did not replicate our results in an independent cohort; to confirm the validity of our results, we replicated our findings in two different target tissues (pancreatic islets and muscle).

In our study, we measured the gene expression profile of whole blood. While this is the tissue one would want to identify a biomarker in, it should not be confounded by the composition of blood cell subtypes. To adjust for this confounding effect, we estimated and measured the fraction of the five major cell types in blood and adjusted for these cell fractions in the model.

Altogether, while gene expression levels are interesting blood biomarkers for poor glycaemic control, our study suggests that gene expression levels in whole blood reflect current glycaemic state, but are not necessarily predictive of future glycaemic state. The genes identified provide an important insight into the link between poor glycaemic control and altered expression of cell cycle and immune pathways in blood, which, for some genes, also extends to the target tissues muscle and pancreas.

## Electronic supplementary material

ESM (PDF 422 kb)

ESM Table 1 (XLSX 3077 kb)

## Abbreviations

DCS: Diabetes Care System
e(GFR): Estimated (GFR)
FDR: False-discovery rate
GEO: Gene Expression Omnibus
PBMC: Peripheral blood mononuclear cell
