# Supplementary material for: HbA1c is associated with altered expression in blood of cell cycle- and immune response-related genes
Source: Diabetologia. 2017 Nov 20;61(1):138–46. doi: 10.1007/s00125-017-4467-0 (PMC6448931; doi:10.1007/s00125-017-4467-0)
Supplement: Supplementary file 1 — (PDF 422 kb) [file 125_2017_4467_MOESM1_ESM.pdf]

Electronic supplemental material

ESM Table 1, Characteristics of overall cohort, those within the biobank and the subsets for RNA and RNA-seq.

	**Entire population (2013)**		**Population in biobank (2013)**		**RNA population**		**RNA-seq population**	
	**(n=8063)**		**(n=4921)**		**(n=1033)**		**(n=391)**	
	Median	*IQR*	*Median*	*IQR*	*Median*[1]	*IQR*	*Median*[1]	*IQR*
Age (years)	67.5	[60.0-75.3]	67.1	[59.9-74.2]	65.0	[57.7-72.4]	64.0	[57.3-70.0]
Sex (% female)	47.3		44.5		43.4		41.2	
Diabetes duration (years)	6.9	[3.2-12.0]	7.6	[4.1-12.5]	4.5	[2.5-8.7]	3.7	[2.1-5.5]
Glucose (mmol/l)	7.8	[6.8-9.0]	7.8	[6.9-9.1]	7.8	[7.0-8.9]	7.9	[7.2-9.1]
HbA_{1c} (%)	6.5	[6.1-7.1]	6.5	[6.1-7.1]	6.4	[6.0-7.0]	6.4	[6.0-7.0]
HbA_{1c} (mmol/mol)	47	[43-54]	47	[43-54]	47	42-53]	47	[42-53]
BMI (kg/m^2)	29.2	[26.3-33.0]	29.2	[26.5-32.9]	29.5	[26.5-33.0]	29.5	[26.4-33.0]
LDL (mmol/l)	2.3	[1.8-3.0]	2.3	[1.8-2.9]	2.3	[1.8-3.0]	2.3	[1.8-2.9]
HDL (mmol/l)	1.2	[1.0-1.5]	1.2	[1.0-1.5]	1.2	[1.0-1.5]	1.2	[1.0-1.5]
Triacylglycerol (mmol/l)	1.5	[1.1-2.1]	1.5	[1.1-2.1]	1.5	[1.1-2.1]	1.6	[1.1-2.2]
Systolic BP (mmHg)	139	[126-155]	138	[126-154]	134	[124-152]	134	[124-152]
Diastolic BP (mmHg)	78	[73-84]	78	[73-84]	78	[74-84]	80	[75-85]
eGFR (ml/min)	81.0	[67.2-95.2]	81.3	[67.8-95.2]	83.0	[70.5-96.4]	85.8	[73.4-98.5]
Metformin use (%)	68.5		70.5		71.2		89.5	
SU use (%)	29.9		31.9		29.9		11.8	
Insulin use (%)	23.3		24.5		17.1		12.0	

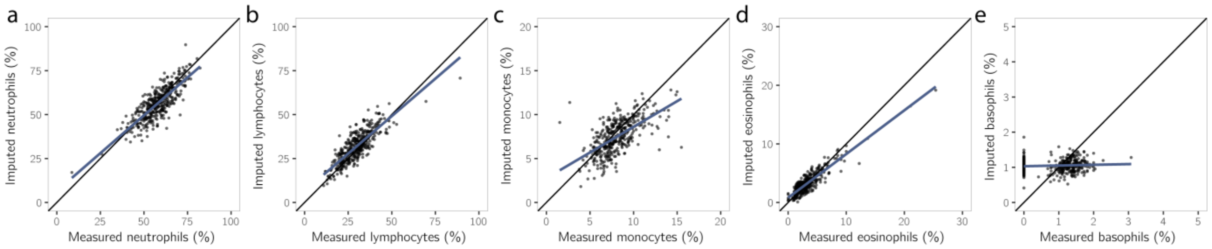

ESM Fig. 1. Measured blood cell percentages against imputed blood cell percentages. **a** Neutrophils, r = 0.81. **b** lymphocytes, r = 0.83. **c** monocytes, r = 0.64. **d** eosinophils, r = 0.91. **e** basophils, r = 0.08.

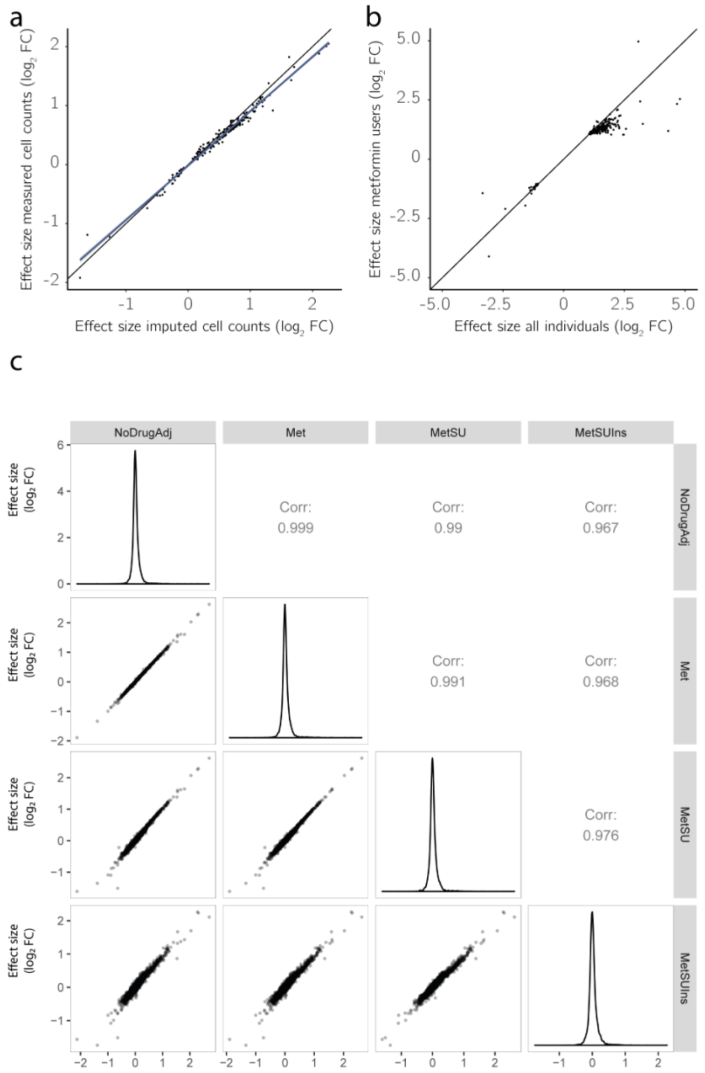

ESM Fig. 2. **a** Comparison of effect size ($\log_2$ fold change) with imputed cell counts and measured cell counts. Pearson's correlation between two models: r = 0.99. **b** Comparison of effect size of the model on all individuals and a stratified analysis on metformin users only. Pearson's correlation between two models: r = 0.94. **c** Comparison of effect size of models 1) without adjustment for medication usage and with for 1) metformin, 2) metformin and SU and 3) metformin, SU and insulin. FC, fold change; Corr, correlation; NoDrugAdj, no adjustment for drug use; Met, adjustment for metformin only; MetSU, adjustment for metformin and sulfonylureas; MetSUIns, adjustment for metformin, sulfonylureas and insulin.

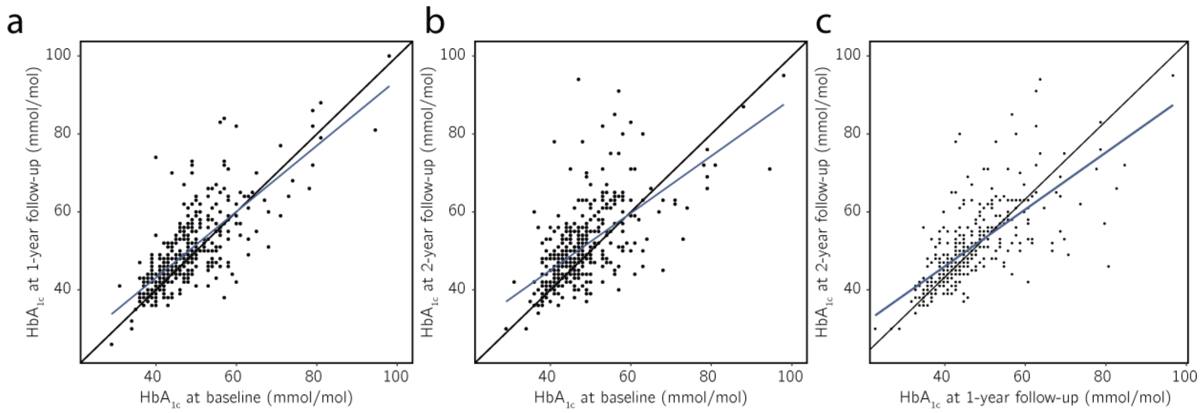

ESM Fig. 3. Correlation of HbA1c between successive years. **a** Baseline HbA$_{1c}$ vs. 1-year follow-up HbA$_{1c}$, n = 370, r = 0.76, $p < 0.0001$. **b** Baseline HbA$_{1c}$ vs. 2-year follow-up HbA$_{1c}$, n = 360, r = 0.64, $p < 0.0001$. **c** 1-year follow-up HbA$_{1c}$ vs. 2-year follow-up HbA$_{1c}$, n = 354, r = 0.74, $p < 0.0001$.

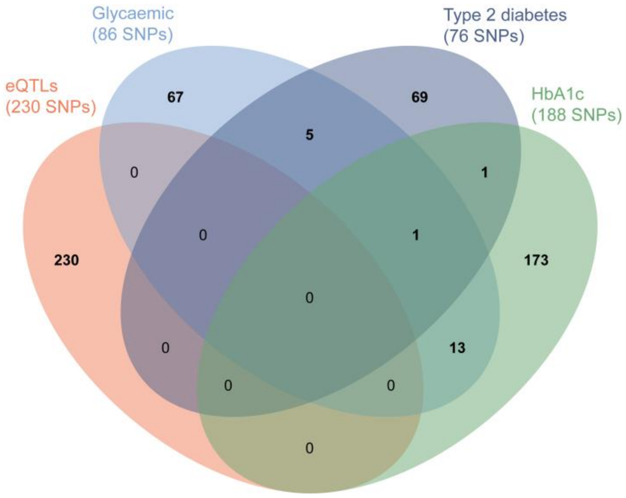

ESM Fig. 4. Overlap between 239 eQTL-SNPs and known diabetes-related traits.
